# Supplementary material for: Neural effects of transcranial magnetic stimulation at the single-cell level
Source: Nat Commun. 2019 Jun 14;10:2642. doi: 10.1038/s41467-019-10638-7 (PMC6572776; doi:10.1038/s41467-019-10638-7)
Supplement: Supplementary file 1 — Supplementary Information [file 41467_2019_10638_MOESM1_ESM.pdf]

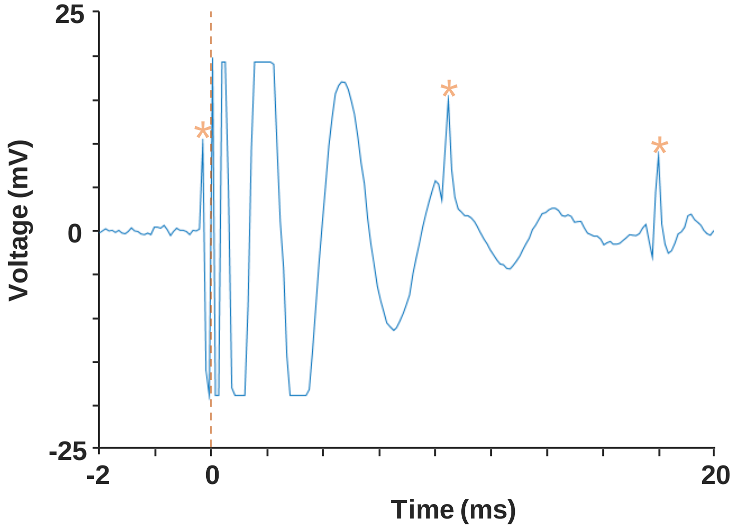

Supplementary Figure 1. TMS artifact. Voltage graph showing the TMS-evoked artifact on a single stimulation trial. By detecting the peaks we were able to isolate individual spikes (indicated in the graph with an orange asterisk) recorded before, during and after TMS. On an average session, the artifact duration lasted about 10 ms. However, the frequency and intensity of the evoked artifact evolved during this period, decaying towards the end, so that in the last phase it was possible to record cellular activity, with spikes overriding the mechanical noise.

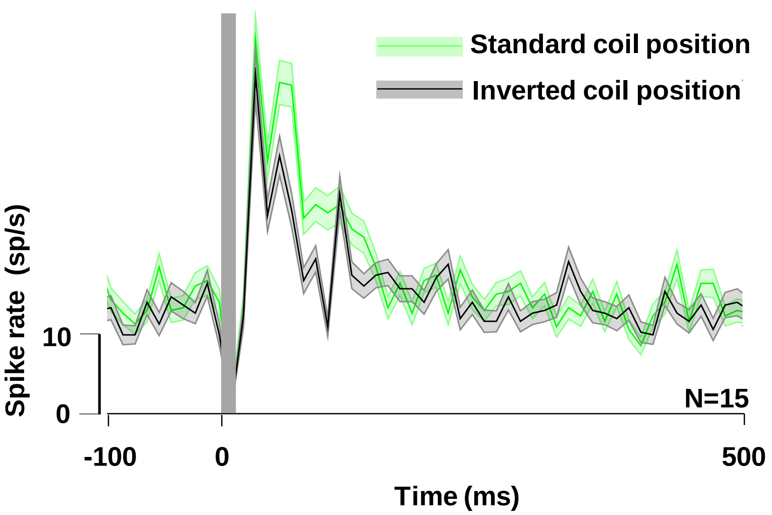

Supplementary Figure 2. TMS effect to the coil inversion. Comparative graph showing the average spike rate of a subset of neurons (N=15) recorded with both TMS applied inducing a posterior-anterior (PA) current flow (standard coil position; green) and an anterior-posterior (AP) current flow (inverted coil position: -180 deg rotation of the coil-; black). Shading represents ± the standard error.

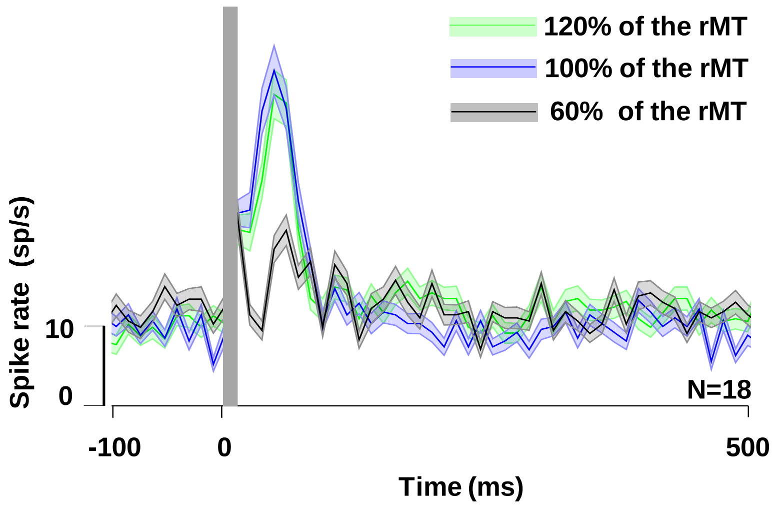

Supplementary Figure 3. Threshold analysis: comparison of the TMS effect to different stimulation intensities. Average spike rate of all neurons recorded in a parallel control study (N=18) aimed to determine the optimal stimulation intensity. After proper isolation, we recorded the activity of the same neurons to single-pulse TMS applied at three different stimulation intensities: 120% of the rMT (high stimulation; green), 100% of the rMT (moderate stimulation; blue) and 60% of the rMT (low stimulation; black). Shading equals ± the standard error.

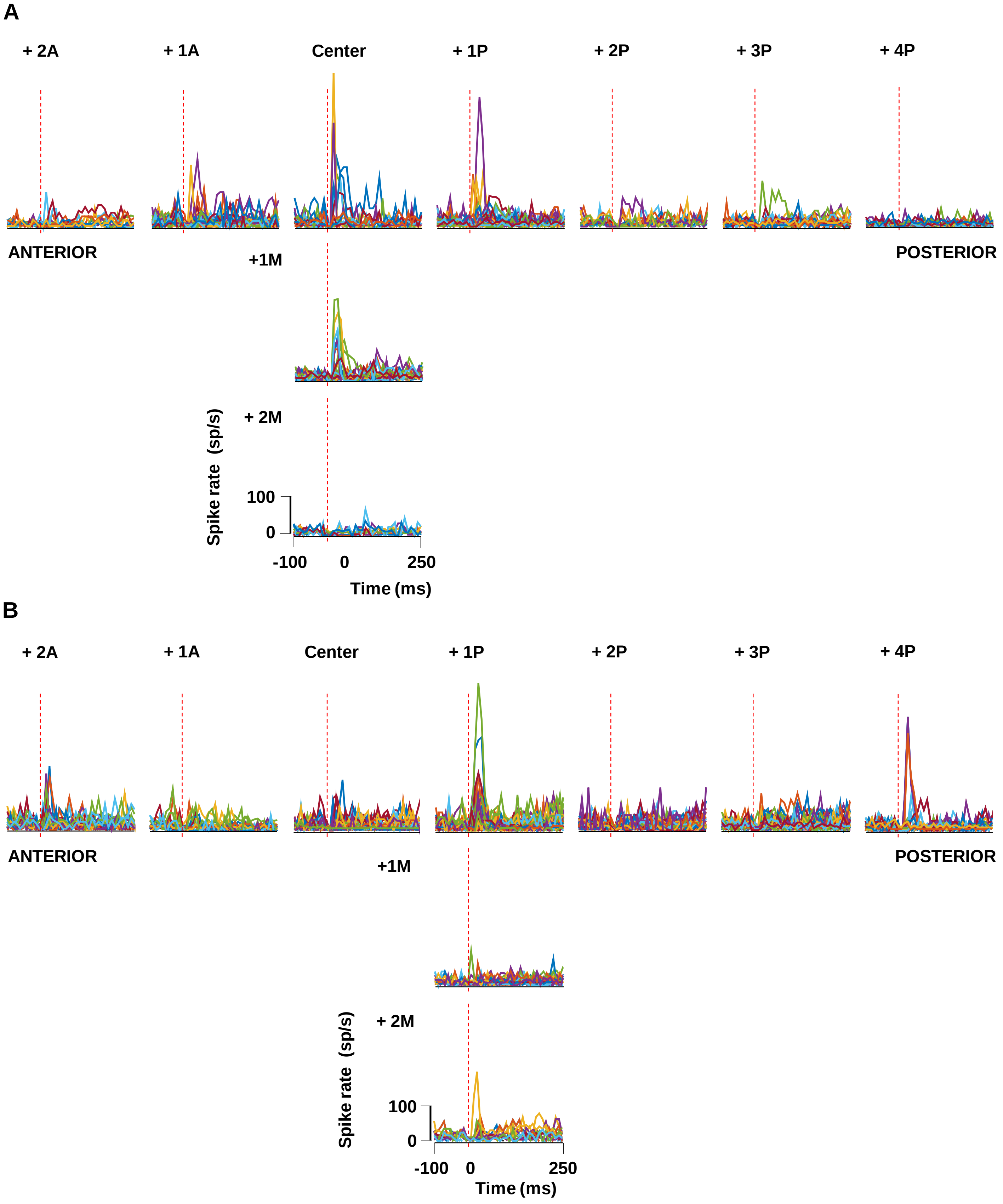

Supplementary Figure 4. Neuronal variability across PFG. A: Spatial map detailing the TMS-induced activity across grid positions (from 2 mm anterior to 4 mm posterior to the center of stimulation; and 0 to 2 mm medial to the center of stimulation) in monkey Y. For each individual graph, colored lines indicate the net response of a single neuron recorded at a particular position. TMS delivery is indicated by a dashed red line, occurring in this case at 'light' onset. **B**: Spatial map in monkey P. Same conventions as in A. For both animals, some neurons outside the center of stimulation were also responsive to TMS. However, on average, the number of affected neurons decreased considerably for positions located only 1 mm away from the center of the coil.

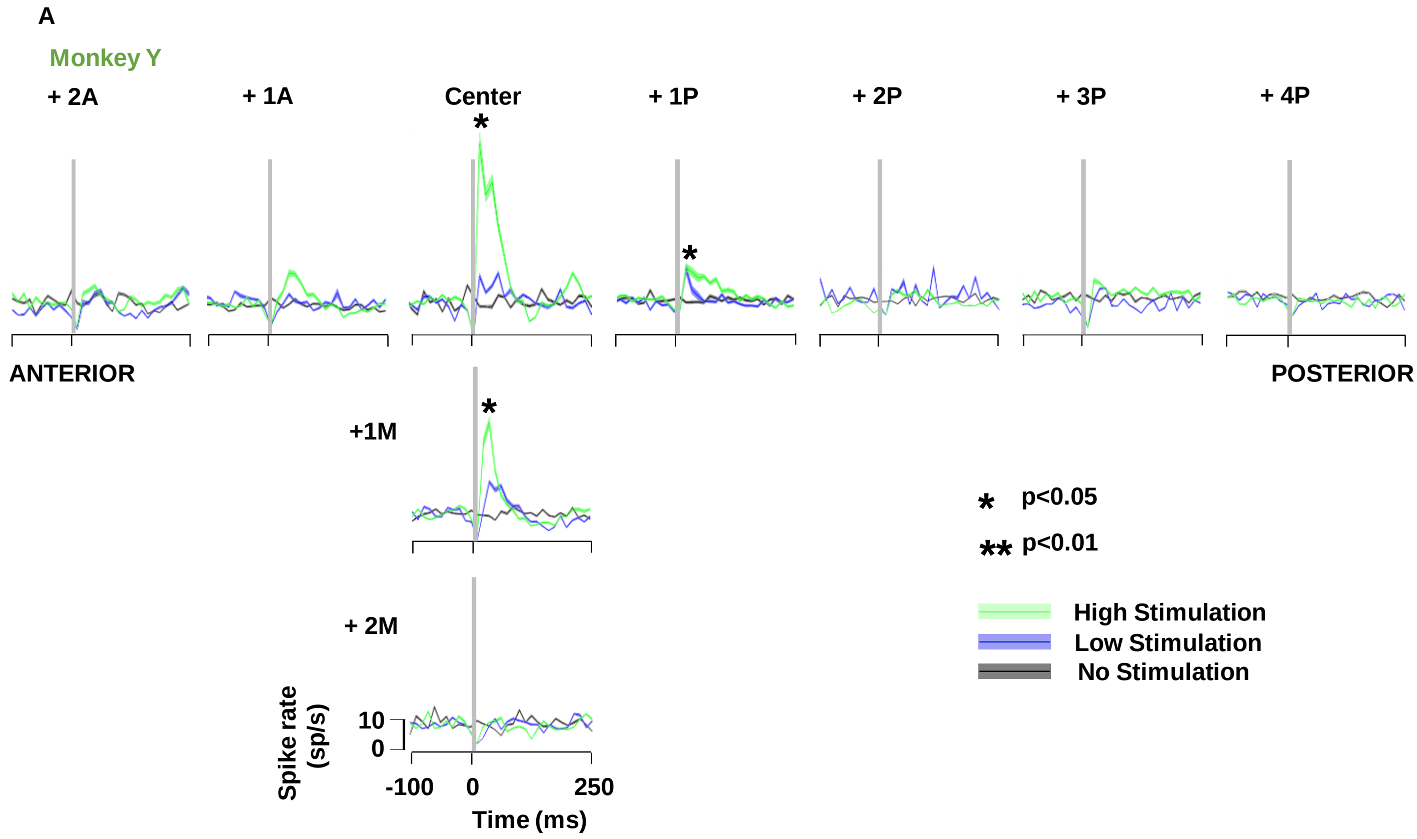

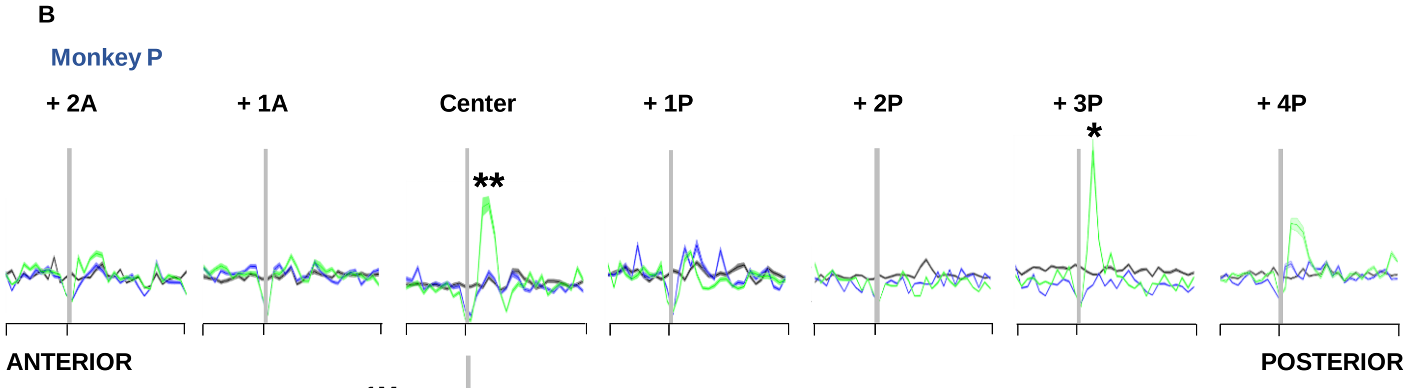

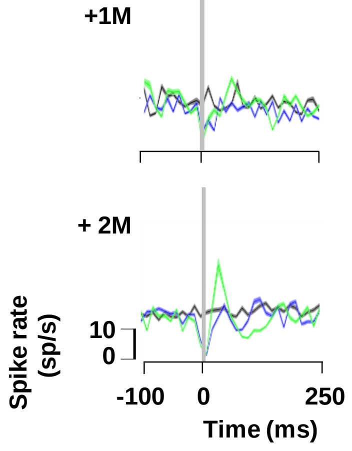

Supplementary Figure 5. Complete map of the spatial spread of the TMS effect, average responses. A: Spatial spread in monkey Y. Average spike rate of all neurons collected at the center of stimulation and neighboring mapped grid positions, extending over 7 mms in the anterior-posterior axis and 3 mms in the mediolateral axis. The figure is arranged in a T-shape to summarize the activity maps obtained for high stimulation (green: 120% rMT), low stimulation (blue; 60% rMT) and no stimulation (black) when TMS was applied at 'light' onset. Asterisks indicate statistical strength (two-sided Wilcoxon ranksum test; *= p ≤ 0.05; **= p ≤ 0.01). As for Figure 3, shading indicates ± the standard error. **B**: Spatial spread in monkey P. Same conventions as in A.

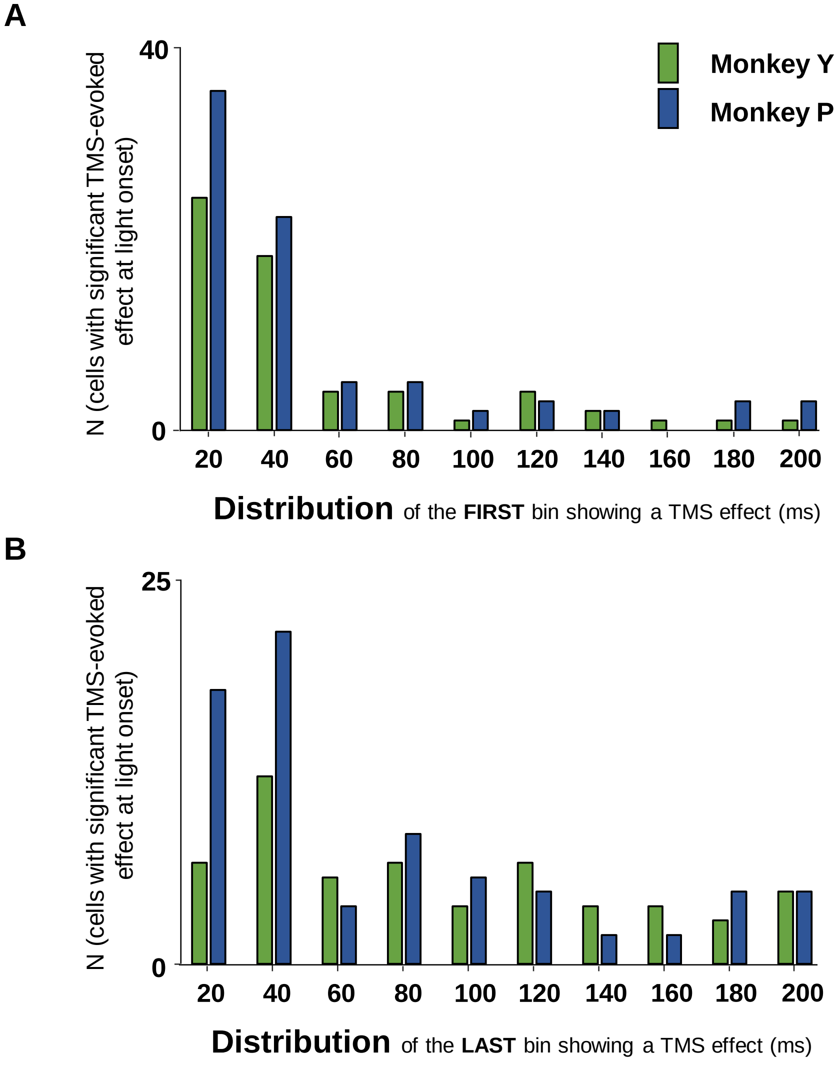

Supplementary Figure 6. Latency and duration of the TMS effects on neuronal activity. **A**: Bin-by-bin analyses of the TMS effect: latency. Distribution of the first bin (bin size=20 ms) showing a significant TMS effect across all positions tested in the two animals (green: monkey Y; blue: monkey P). The first bin analyzed extended from 10 to 30 ms after TMS onset; the median values were identical across subjects (40 ms). **B**: Bin-by-bin analyses of the TMS effect: duration. For the same analyses, distribution of the last significant bin (median value for both monkeys = 40 ms). Same conventions as in A.

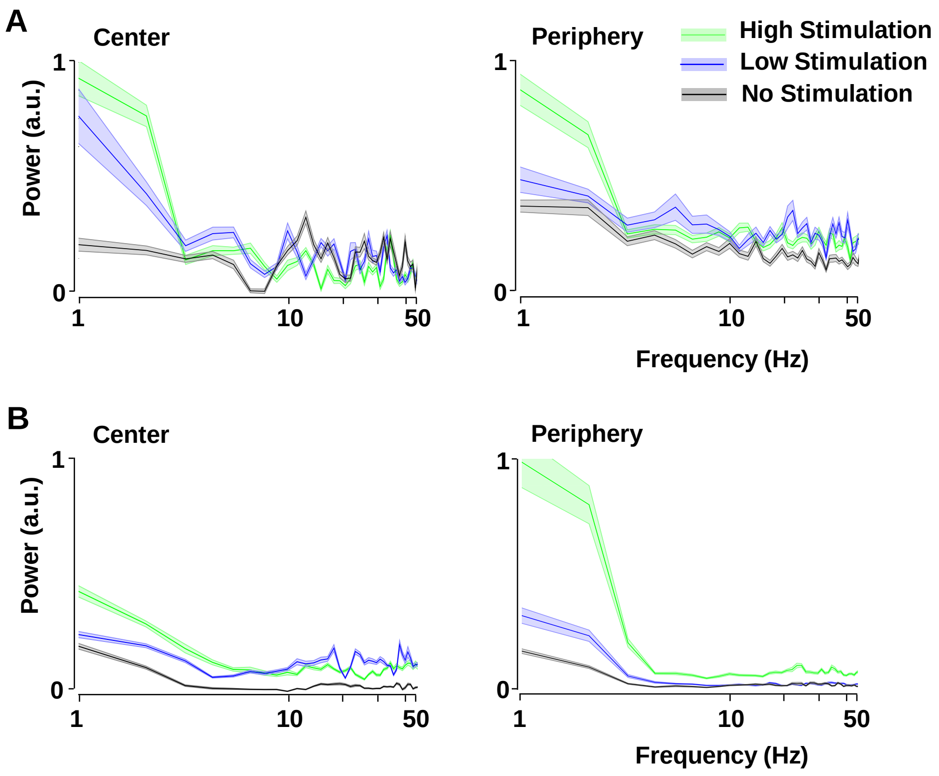

Supplementary Figure 7. Spike oscillations. A: Spike oscillations analysis in monkey Y. We performed spectral analysis on the single-units using a Hanning-tapered Fourier transformation, and looked at the oscillatory activity induced at both the center of stimulation (left panel) and the periphery (right panel; combined response of all neurons recorded outside the center) in response to high TMS stimulation (120% of the RMT; green) compared to low stimulation (60% of the rMT; blue) and no stimulation (black). The shading in the graph represents ± the standard error. **B:** Same analyses for monkey P.

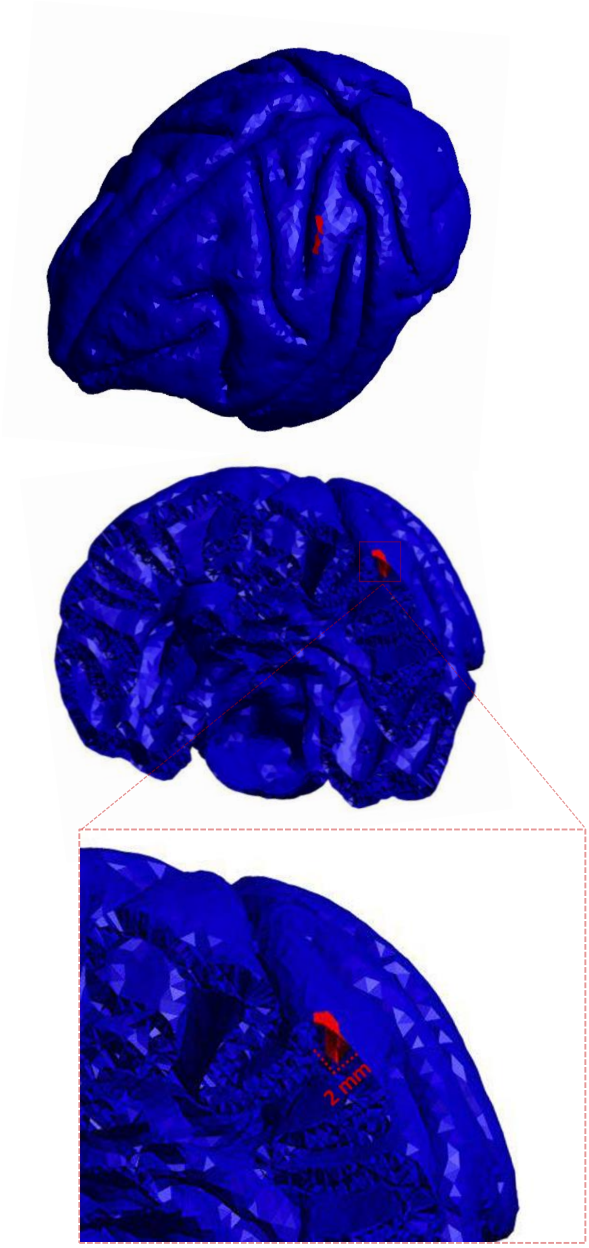

Supplementary Figure 8. Simulated stimulation area. The region in red corresponds to the volume of cortex (approximately 2 by 2 by 2 mm) showing TMS-evoked activity in our electrophysiological recordings. The electric field in this region showed values between 95-100%.

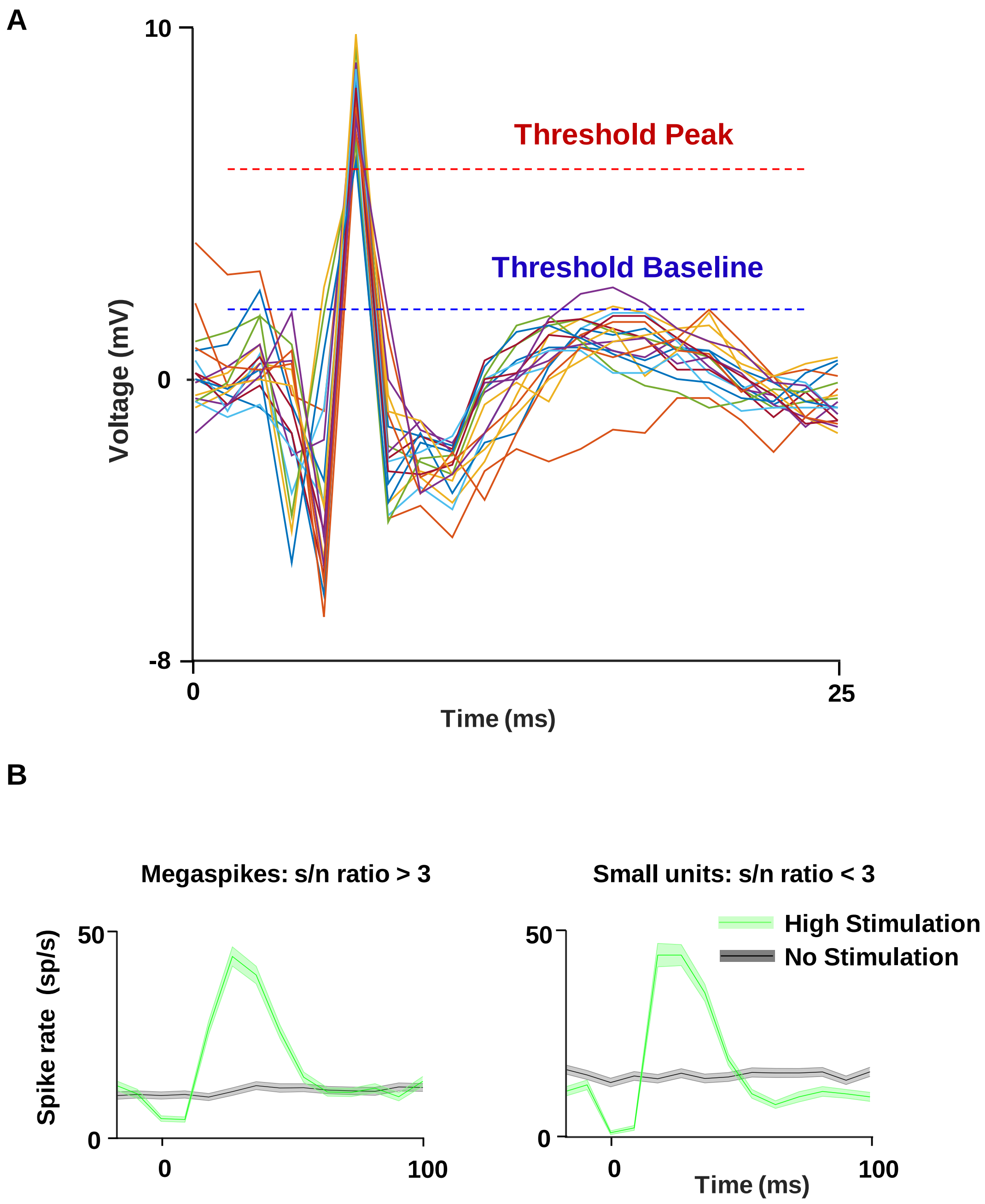

Supplementary Figure 9. Offline spike sorting. **A**: Voltage diagram showing the analyses performed on single spikes to differentiate neuronal size (single- and multi-unit activity). For each individual isolated spike (color lines), a baseline (noise) threshold and a peak threshold (signal= 3 x baseline) were assigned to calculate the signal-to-noise ratio (SNR). All neurons showing an SNR equal or higher than 3 were classified as single-units or megaspikes. In contrast, neurons with an SNR lower than 3 were considered small units, which were often associated to multi-unit recordings. **B**: Average spike rate (shading illustrates ± the standard error) obtained for all neurons classified as megaspikes (left) and small-units (right), recorded in both animals at the center of stimulation. A direct comparison of the high stimulation (green: 120% of the rMT) and no stimulation (black) conditions in both populations evidenced that the evoked TMS effect was similar across neuronal types (size).

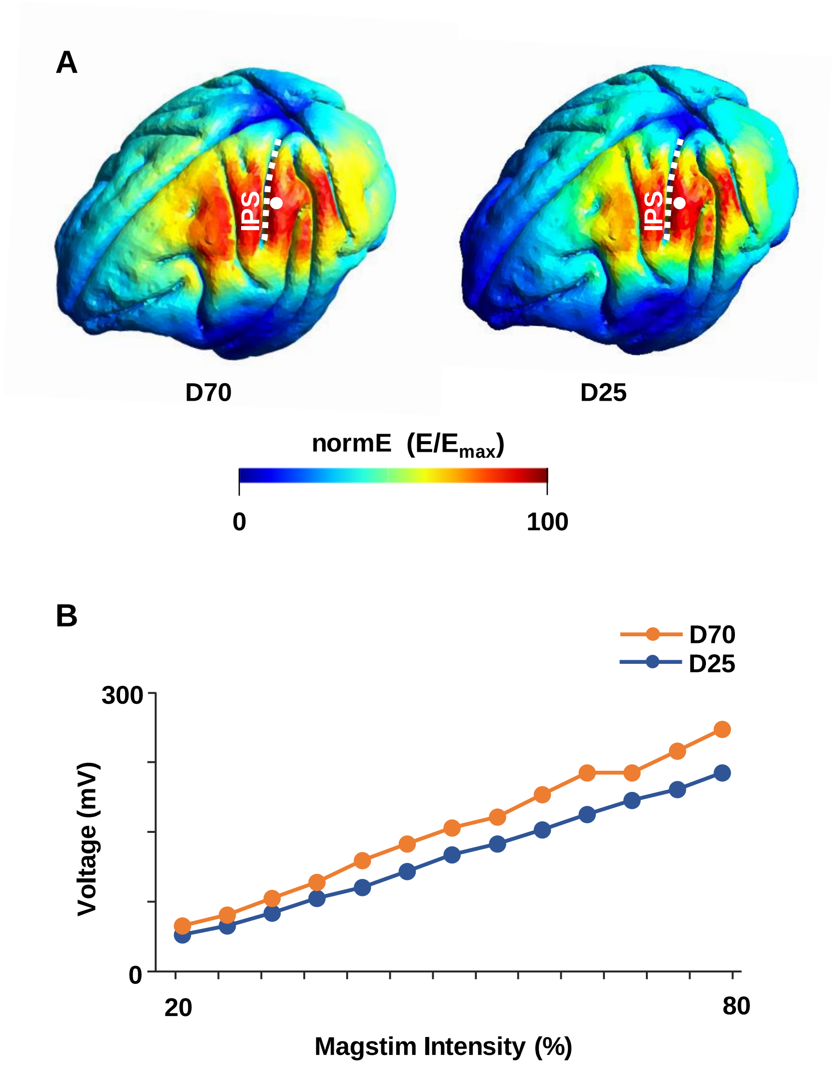

Supplementary Figure 10. simNIBS model and coil measurements. A: TMS-induced electric field as modelled with simNIBS. Rotated models of the brain showing the normalized electric field distribution (spatial spread) calculated for two different coils: D70 (left) and D25 (right). The white dot indicates the center of stimulation (center of the coil). **B**: Output voltage measurements taken with the D70 and the D25 coils at a distance of 15 mm. Using a home-made mini-coil, we took readings of the evoked voltage by applying single-pulses at different intensities (20-80% of the Magstim Rapid).
