# Supplementary material for: Neural effects of transcranial magnetic stimulation at the single-cell level
Source: Nat Commun. 2019 Jun 14;10:2642. doi: 10.1038/s41467-019-10638-7 (PMC6572776; doi:10.1038/s41467-019-10638-7)
Supplement: Supplementary file 3 — Reporting Summary [file 41467_2019_10638_MOESM3_ESM.pdf]

nature research

Corresponding author(s): DBPR; NCOMMS-18-25867A

Last updated by author(s): 23/04/2019

Reporting Summary

Nature Research wishes to improve the reproducibility of the work that we publish. This form provides structure for consistency and transparency in reporting. For further information on Nature Research policies, see Authors & Referees and the Editorial Policy Checklist.

Statistics

For all statistical analyses, confirm that the following items are present in the figure legend, table legend, main text, or Methods section.

n/a	Confirmed	
☐	☒	The exact sample size (n) for each experimental group/condition, given as a discrete number and unit of measurement
☐	☒	A statement on whether measurements were taken from distinct samples or whether the same sample was measured repeatedly
☐	☒	The statistical test(s) used AND whether they are one- or two-sided<br>*Only common tests should be described solely by name; describe more complex techniques in the Methods section.*
☒	☐	A description of all covariates tested
☐	☒	A description of any assumptions or corrections, such as tests of normality and adjustment for multiple comparisons
☒	☐	A full description of the statistical parameters including central tendency (e.g. means) or other basic estimates (e.g. regression coefficient) AND variation (e.g. standard deviation) or associated estimates of uncertainty (e.g. confidence intervals)
☒	☐	For null hypothesis testing, the test statistic (e.g. F, t, r) with confidence intervals, effect sizes, degrees of freedom and P value noted<br>*Give P values as exact values whenever suitable.*
☒	☐	For Bayesian analysis, information on the choice of priors and Markov chain Monte Carlo settings
☒	☐	For hierarchical and complex designs, identification of the appropriate level for tests and full reporting of outcomes
☒	☐	Estimates of effect sizes (e.g. Cohen's d, Pearson's r), indicating how they were calculated

Our web collection on statistics for biologists contains articles on many of the points above.

Software and code

Policy information about availability of computer code

Data collection	Magstim 200 and Magstim Rapid Stimulators (Magstim, UK); hydraulic microdrive (FHC); BAK Electronics preamplifier (Model A-1)
Data analysis	MATLAB (MathWorks, Massachusetts, USA)

For manuscripts utilizing custom algorithms or software that are central to the research but not yet described in published literature, software must be made available to editors/reviewers. We strongly encourage code deposition in a community repository (e.g. GitHub). See the Nature Research guidelines for submitting code & software for further information.

Data

Policy information about availability of data

All manuscripts must include a data availability statement. This statement should provide the following information, where applicable:

- Accession codes, unique identifiers, or web links for publicly available datasets
- A list of figures that have associated raw data
- A description of any restrictions on data availability

All datasets are available at Dryad (doi:10.5061/dyrad.g54381n). Additional modified scripts can be accessed upon request. Similarly, all simNIBS parameters and brain volumes are available from the corresponding authors on request.

Field-specific reporting

Please select the one below that is the best fit for your research. If you are not sure, read the appropriate sections before making your selection.

☒ Life sciences ☐ Behavioural & social sciences ☐ Ecological, evolutionary & environmental sciences

Life sciences study design

All studies must disclose on these points even when the disclosure is negative.

Sample size	We recorded the activity of 538 parietal neurons (476 in the standard experiment and 62 in a control experiment; M1: 172, M2: 304) in two rhesus monkeys.To compare datasets with a small sample size we have used nonparametric or distribution-free tests, which reduce the effect of the possible outliers and eliminate the assumption of normality (the underlying distribution is normal). Repeated measures (trials) also guarantee greater statistical power, allowing a smaller sample size.
Data exclusions	In our study, no data have been excluded.
Replication	Our results were highly replicable both across neurons and experimental subjects.
Randomization	N/A
Blinding	N/A

Reporting for specific materials, systems and methods

We require information from authors about some types of materials, experimental systems and methods used in many studies. Here, indicate whether each material, system or method listed is relevant to your study. If you are not sure if a list item applies to your research, read the appropriate section before selecting a response.

Materials & experimental systems

n/a	Involved in the study	
☒	☐	Antibodies
☒	☐	Eukaryotic cell lines
☒	☐	Palaeontology
☐	☒	Animals and other organisms
☒	☐	Human research participants
☒	☐	Clinical data

Methods

n/a	Involved in the study	
☒	☐	ChIP-seq
☒	☐	Flow cytometry
☒	☐	MRI-based neuroimaging

Animals and other organisms

Policy information about studies involving animals; ARRIVE guidelines recommended for reporting animal research

Laboratory animals	Macaca mulatta; N=2 ; Gender: male.
Wild animals	N/A
Field-collected samples	N/A
Ethics oversight	All experimental procedures were performed in accordance with the National Institutes of Health Guide for the Care and Use of Laboratory Animals and the EU Directive 2010/63/EU, and were approved by the Ethical Committee at KU Leuven.

Note that full information on the approval of the study protocol must also be provided in the manuscript.
